# Proteochemometric Modeling of the Antigen-Antibody Interaction: New Fingerprints for Antigen, Antibody and Epitope-Paratope Interaction

**DOI:** 10.1371/journal.pone.0122416

**Published:** 2015-04-22

**Authors:** Tianyi Qiu, Han Xiao, Qingchen Zhang, Jingxuan Qiu, Yiyan Yang, Dingfeng Wu, Zhiwei Cao, Ruixin Zhu

**Affiliations:** 1 Department of Bioinformatics, School of Life Sciences and Technology, Tongji University, Shanghai 200092, China; 2 Department of Computer Science, University of Helsinki, Helsinki, FI-00014, Finland; 3 Shanghai Center for Bioinformation Technology, Shanghai 201203, China; 4 School of Pharmacy, Liaoning University of Traditional Chinese Medicine, Dalian 116600, Liaoning, China; Vrije Universiteit Brussel, BELGIUM

## Abstract

Despite the high specificity between antigen and antibody binding, similar epitopes can be recognized or cross-neutralized by paratopes of antibody with different binding affinities. How to accurately characterize this slight variation which may or may not change the antigen-antibody binding affinity is a key issue in this area. In this report, by combining cylinder model with shell structure model, a new fingerprint was introduced to describe both the structural and physical-chemical features of the antigen and antibody protein. Furthermore, beside the description of individual protein, the specific epitope-paratope interaction fingerprint (EPIF) was developed to reflect the bond and the environment of the antigen-antibody interface. Finally, Proteochemometric Modeling of the antigen-antibody interaction was established and evaluated on 429 antigen-antibody complexes. By using only protein descriptors, our model achieved the best performance ($R2=0.91,Q_{test}^{2}=0.68$) among peers. Further, together with EPIF as a new cross-term, our model ($R2=0.92,Q_{test}^{2}=0.74$) can significantly outperform peers with multiplication of ligand and protein descriptors as a cross-term ($R2\leq 0.81,Q_{test}^{2}\leq 0.44$). Results illustrated that: 1) our newly designed protein fingerprints and EPIF can better describe the antigen-antibody interaction; 2) EPIF is a better and specific cross-term in Proteochemometric Modeling for antigen-antibody interaction. The fingerprints designed in this study will provide assistance to the description of antigen-antibody binding, and in future, it may be valuable help for the high-throughput antibody screening. The algorithm is freely available on request.

## Introduction

Antigen-antibody interaction is an important and fundamental biochemical function in immune system. By recognizing the epitope area on the surface of protein antigen, antibodies secreted by B-cell are able to interact with those invasive antigens and then neutralize them to keep our body safe [1,2]. However, for the new emerging antigens caused by mutation, previous antibody may not work effectively due to the antigenicity variance. Since the mechanism of antigen-antibody interaction remains elusive, when a new antigen emerges, experimental methods are most frequently used to test whether the previous antibody or antiserum can still recognize the new antigen or not [3], or to produce functional antibody molecules corresponding to the antigen through mass clonal cell screening [4]. As a special protein-protein interaction, antigen-antibody interaction occurs neither in the whole protein nor in the entire surface, but in the specific “binding site” [5]. For the antigen-antibody interaction, these specific “binding site” can be called as “epitope-paratope interaction site” [1,2]. It has been frequently reported that one or several mutation in “binding site” often lead to large binding affinity changes [3,6,7,8,9]. This may correspond to two interesting phenomenon in antigen-antibody interaction, one is that antigens may change a few amino acids to produce a new epitope through continual antigenic drift [10]; the other one is that antibodies can recognize millions of different antigens through minor amino acid changes in paratope area [11]. Both “antigenic drift” mutations in epitopes and “adaptive” mutations in paratopes are caused by amino acid sequence or structure variations. Despite of the high specificity between antigen and antibody binding, different studies have showed that similar epitopes can still be recognized or cross-neutralized by the same antibody [12] or biological trigger [13]. Therefore, how to accurately characterize the interface of “epitope-paratope interaction” and how to handle multi-target screening problems is the key issue to study the mechanisms of interaction between those biological macromolecules [5].

Till now, many methods have been developed to characterize the interface features of protein, which can be roughly divided into three categories: 1) Geometry-based [14,15,16]; 2) Energy-based [17] and 3) Signature-based [18,19] methods. “Geometry-based” methods contain three aspects: “amino acid-based” [14], “atom-based” [15] and “Geometric & Physical-chemical-based” [16] method. These kinds of method utilize three-dimensional coordinates of atoms, pseudo-atoms and residues to superimpose two structures and quantify their similarity. “Energy-based” methods refer to those decomposition methods after molecular dynamic simulations. Those methods can decompose the binding free energy in the interaction interface into specific residues, and quantitatively characterize the contribution of various residues for the entire protein-protein interaction [20]. Compared to those two above methods, “Signature-based” methods [18,19,21] do not require numerous computing resources and precise three-dimensional coordinate information, which may make it more robust when dealing with slight structural changes occurs in the “binding site” [5].

These methods greatly promoted development for “protein binding site” analysis. However, above methods exist several limitations in the case of “epitope-paratope interaction”: “Geometric-based” methods and “Signature-based” methods only derive relevant features either from receptor side or ligand side without considering interaction features. This is not able to completely describe the features of “epitope-paratope interaction”. As for the “energy-based” methods, molecular dynamic simulation process took the information of interaction into account, however, due to the time-consuming simulations, it is not able to achieve high-throughput screen analysis. Moreover, the “energy-based” methods may often unable to extract geometric features in the interaction interface, which makes it can only be used to build explanatory models. Therefore, developing a new descriptor for “protein binding site”, which can reflectboth spatial geometric features and interaction forces with robustness, accuracy and operational efficiency, is highly desired. A recent idea of “interaction fingerprint” developed in the area of drug design makes it possible to analyze the interaction between two molecular structures [22]. By taking features of antigen-antibody interaction into consideration, a new set of epitope-paratope interaction fingerprint (EPIF) has been firstly generated to describe the antigen-antibody interaction. Meanwhile, a new set of protein descriptors has been established to describe the residue layout and physical-chemical features for both antigen and antibody proteins.

As an extension of the quantitative structure-activity relationship (QSAR) methods, Proteochemometric (PCM) Modeling has been widely used to study the cross-interactions between a series of ligands and a series of receptors [23,24]. Different from QSAR, PCM contains information from both the ligand and the target descriptors to correlate with activity data. Moreover, an additional term ‘cross-term’ was introduced to describe interaction features and most of the previous studies defined the cross-terms of PCM model as the Multiplication of Ligand and Protein Descriptors (MLPD) [25,26]. It is worthy of compliment that MLPD contains information from both side of the interaction interface, which can be considered as candidates of cross-term. However, MLPD is generated by the multiplication of ligand and protein descriptors, which has higher time-complexity (*n* ^2^) than single side descriptors (*n*), also the significance of MLPD is not easy to interpret. Thus, our new invented epitope-paratope interaction fingerprints (EPIF) which describes the antigen-antibody interaction can be used as “cross-terms” to address this issue. By combining our new protein fingerprint with EPIF, Proteochemometric Modeling was constructed to simulate the relationship between multiple antigen and antibody proteins in this study.

## Results and Discussion

### Kernel Selection

Our PCM modeling was performed by employing support vector regression (SVR) methods with different kernels. As a widely used regression model, SVR has a number of advantages over the conventional linear regressions, especially for its robustness to avoid over-fitting [27,28]. By the use of non-linear kernel, SVR projects the data into a high-dimensional space and constructs a set of hyperplanes in it for regression. The construction of learning machine is based on how the inner-product kernel is generated. Therefore, the selection of the kernel function is very important. In our study, four commonly used kernels (Table 1) were implemented in SMOreg of Weka (version 3.7) with default parameters. Previous studies indicated that kernel may perform differently on different datasets, and the adaptation of kernels were based on the type of the dataset [29]. In our PCM modeling, 10-fold cross-validation was evaluated on all four kernels to select effective kernel functions. The cross-validation results ($Q_{CV}^{2})$ of each kernel with different combination of fingerprints were listed in Table 2. The results showed that Normalized Poly Kernel function obtains better predictive ability than the other three kernel functions. Therefore, Normalized Poly Kernel function was selected for PCM modeling and performance evaluation.

**Table 1:** Summary of Kernels.

Type of Kernels	Functions
**Normalized Poly Kernel**	$k\left(x,y\right)={\left(x^{T}y+c\right)}^{d}/\sqrt{\left(x^{T+1}+y^{T+1}\right)}$
**Polynomial Kernel**	*k*(*x*,*y*) = (*x* ^*T*^ *y* + *c*)^*d*^
**Puk**	kx,y=1[1+(2||x−y||22(1ω)−1σ)2]ω
**RBF Kernel**	*k*(*x*,*y*) = exp(−*γ*‖*x*–*y*‖^2^)

**Table 2:** $Q_{CV}^{2}$ of our three fingerprint combinations with different SVR methods and kernels.

Fingerprint\Kernel	Normalized Poly Kernel	Polynomial Kernel	Puk	RBF Kernel
Fab-Fag-EPIF^a^	**0.52**	0.35	0.40	0.51
Fab-Fag-MLPD^b^	**0.49**	0.36	0.26	0.49
Fab-Fag^c^	**0.47**	0.31	0.38	0.43
Average	**0.49**	0.34	0.35	0.48

^a^Models created using antibody fingerprint and antigen fingerprint with EPIF as cross-term

^b^Models created using antibody fingerprint and antigen fingerprint with the multiplication of antibody fingerprint and antigen fingerprint as cross-term

^c^Models created using only antibody fingerprint and antigen fingerprint.

### Development and evaluation of Proteochemometric Modeling

Proteochemometric model with different combination of descriptors were summarized in Table 3. To evaluate the performance of our antigen-antibody interaction fingerprint in Proteochemometric Modeling, three fingerprint combinations (Fab-Fag-EPIF, Fab-Fag-MLPD, Fab-Fag) were tested. Results indicated that Fab-Fag-EPIF obtained better predictive ability than those without cross-terms or those using MLPD as cross-terms. Also, the prediction performance of Fab-Fag-EPIF and Fab-Fag were better than the model with MLPD as cross-terms, which illustrated that the conventional cross-term of MLPD was not only being outperformed by new introduced cross-term of PLIF but also being surpassed by our protein fingerprints without cross-terms.

**Table 3:** Goodness-of-fit (*R* ^2^) and predictive ability ($Q_{test}^{2}$) of the models which were obtained by different model.

Fingerprint\Kernel	*R* ^2^	$Q_{test}^{2}$	*MAE*	*RMSE*	*RAE*	*RRSE*
Fab-Fag-EPIF^a^	0.92	**0.74**	**124.10**	**164.28**	**69.12%**	**69.41%**
Fab-Fag-MLPD^b^	0.99	0.61	139.44	187.92	77.66%	79.39%
Fab-Fag^c^	0.91	**0.68**	**131.17**	**175.30**	**73.06%**	**74.06%**
Sab-Sag^d^	0.79	0.50	137.86	188.21	82.13%	86.26%
Gab-Gag^e^	0.39	0.21	150.17	193.58	94.85%	97.70%
Sab-Sag-EPIF^f^	0.81	0.44	150.11	214.66	84.44%	92.70%
Gab-Gag-EPIF^g^	0.57	0.42	137.56	179.80	86.88%	90.75%
Gab-Gag-MLPD^h^	0.41	0.22	149.66	193.45	94.52%	97.64%

^a^Models created using antibody fingerprint and antigen fingerprint with EPIF as cross-term

^b^Models created using antibody fingerprint and antigen fingerprint with the multiplication of antibody fingerprint and antigen fingerprint as cross-term

^c^Models created using only antibody fingerprint and antigen fingerprint.

^d^Models created using only sequence similarity descriptor of antibody and sequence similarity descriptor of antigen

^e^Models created using only geometry descriptor of antibody and geometry descriptor of antigen

^f^Models created using sequence similarity descriptor of antibody and sequence similarity descriptor of antigen with EPIF as cross-term

^g^Models created using geometry descriptor of antibody and geometry descriptor of antigen with EPIF as cross-term

^h^Models created using geometry descriptor of antibody and geometry descriptor of antigen with the multiplication of antibody descriptor and antigen descriptor as cross-term

The original idea of cross-terms is to add information from both sides of ligand-target interaction [30], which intended to describe the features of the interface between ligand and protein. For protein-protein interaction, especially antigen-antibody interaction, the interface features maybe more related to the interaction forces and environments of the binding site. Thus, interaction fingerprint which is generated from the antigen-antibody complexes and could directly describe the interaction between antigen and antibody from different aspects of important features may be more suitable for cross-terms [31]. Cross-terms calculated by the multiplication of ligand and target descriptors may not be a reliable reflection of the binding side, sometimes performed even worse than those only use fingerprints of both antibody and antigen side [31]. Therefore, it may indicate that, in the case of antigen-antibody recognition, only when a suitable cross-term such as EPIF is used in Proteochemometric Modeling, the model performance can be significantly improved.

### Compared with peers

Existed protein descriptors can be divided into sequence similarity descriptors and geometric structure descriptors [32]. In this study, both sequence similarity descriptor and geometry descriptor were compared with our fingerprints. For sequence similarity descriptor, the amino acid sequences of all the antigen and antibody proteins were retrieved from PDB [33]. BLAST (version2.2.28) was used to calculate sequence identities of all the antigen and antibody structures. Finally, a 429-bit sequence similarity descriptor was obtained. For geometric descriptor, three different aspects were taken into considerations: bond length, bond angel and dihedral angle. 41-bit of protein geometry descriptors were obtained for each antigen-antibody proteins in our dataset. Types of protein geometry descriptors could be seen in S1 Table.

**Fig 1:**
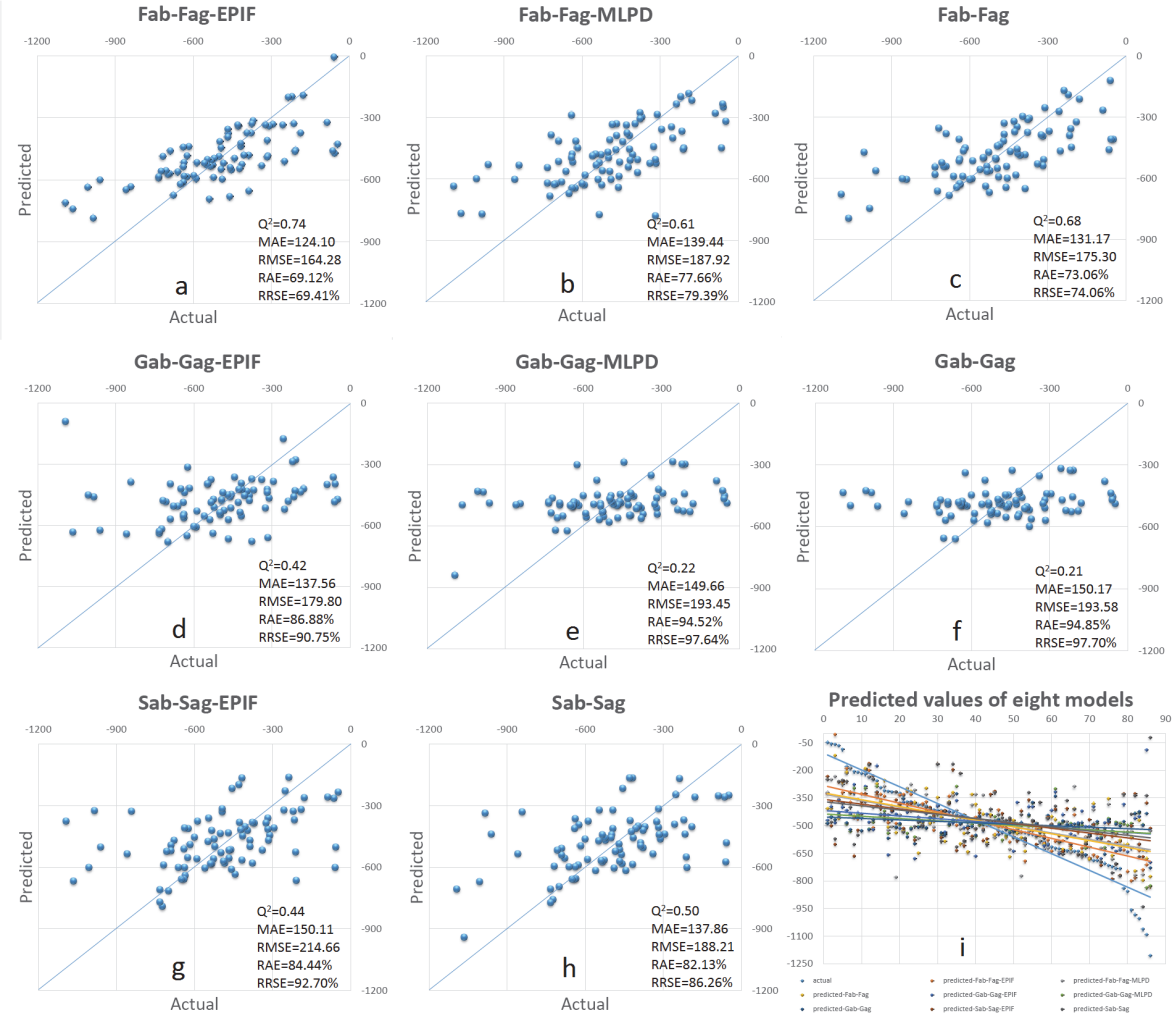
Predicted binding energy of all antigen-antibody in our testing set. Panels a~h represent the predicted value compared with actual value simulated by Hex. Panel i represents the graphical illustrations of the predictive ability of all 8 obtained models with the selected kernel.

The performance of our antigen-antibody fingerprints compared with peers can be found in **Fig 1 and Table 3**. Here, 8 different combinations of descriptor were used to establish the PCM model (The MLPD of sequence similarity descriptors contains 429*429 bits, which were not adopted in this study). For using protein descriptor only, results indicated that the fingerprint of Fab-Fag $\left(R^{2}=0.91,Q_{test}^{2}=0.68\right)$ outperformed other descriptors$\left(R^{2}\leq 0.79,Q_{test}^{2}\leq 0.50\right)$. After added cross-terms, the introducing of EPIF as cross-terms combined with our protein fingerprints (Fab-Fag-EPIF) can achieve the best predictive ability $\left(Q_{test}^{2}=0.74\right)$ among all others$\left(Q_{test}^{2}\leq 0.50\right)$. This demonstrated that, Proteochemometric Modeling with our new invented antigen-antibody structure fingerprint and EPIF may be more appropriate than existed protein sequence similarity descriptors or structure geometric descriptors in the case of antigen-antibody interaction. Results also indicated that the prediction ability of using only the antibody and antigen geometric descriptors (Gab-Gag) is the bottom line of the PCM model as well as the prediction ability of added multiplication of antibody and antigen geometric descriptors as cross-term (Gab-Gag-MLPD). However, by adding EPIF as cross-term to geometric descriptors (Gab-Gag-EPIF), predictive ability can be further increased. On the other hand, the result of sequence similarity descriptors seems performed better than those with EPIF as cross-terms. It might be caused by the fact that sequence similarity descriptor describe the sequence features of protein, but the EPIF focuses on those structure characteristics in the binding interface, so the EPIF can increase the predictive ability of structure descriptors but does not apply well with sequence descriptors.

## Conclusions

Currently, we can only rely on experimental methods to test the binding affinity of mutated antigens with certain antibody or antiserum. Considering the time-consuming experimental methods, computational methods which can accurately describe the antigen-antibody interaction and further help the measurement of binding affinity is highly desired. In this work, a series of protein fingerprint with epitope-paratope interaction fingerprint (EPIF) were firstly introduced and successfully tested on benchmark dataset through Proteochemometric Modeling. The results indicated that our new established protein fingerprint achieved a better predictive ability than peers. In addition, when cross-terms were introduced into Proteochemometric model, the newly established EPIF not only significantly improved the prediction ability, but also outperformed the pervious cross-terms of MLPD. Results also proposed that EPIF as a structure descriptor can increase the predictive performance of the Proteochemometric model based on conventional structure descriptors, but may not be suitable for sequence descriptor. Moreover, our recommended model based on support vector regression with descriptor combination of Fab-Fag-EPIF showed the ability to simulate bonding affinities for antigen-antibody complexes. With known or simulated conformational structures of antigen-antibody complexes, this new established fingerprint will be able to simulate binding affinity, and further, provide assistance for antibody screening.

## Materials and Methods

### Data set

Training and validation dataset of antigen-antibody complexes were extracted from Protein Data Bank [33]. We artificially excluded the inappropriate searching results such as: structures containing only antigen or antibody, T cell epitope-antibody complex structure. Also, structures with low crystalline precision and short sequence length had been excluded to ensure the quality of our dataset. Specific steps and parameters are given as follows:

1. Searching Keywords: antibody, antigen, Fab, Fv, Fc, IgG and immu*
2. Resolution better than 3.0 Å
3. Antigen length with more than 50 residues
4. Two structures share identical sequence and conformational in both epitope and paratope, one of them were removed from our dataset

After these four steps, crystal structures of 429 antigen-antibody complexes including 343 as training data and 86 as testing data were collected. The PDB IDs in our dataset can be found in the Supplementary Data (S2 and S3 Tables).

### Epitope and Paratope determination

For each antigen-antibody complex structure in our dataset, epitope and paratope residues were distinguished by Solvent Accessible Surface Area (SASA) based methods. SASA values were calculated (Naccess V2.1.1) for each residue in antigen-antibody complexes and the single molecule structure with probe radius set as 1.4 Å. Surface residues were those more than 1Å^2^ SASA while those loss in binding of more than 1Å^2^ were classified as epitope on the antigen side and as paratope on the antibody side.

### Interaction energy simulation

To create Proteochemometric models with different descriptors, binding affinity values of each antigen-antibody complex were simulated by Hex [34]. To guarantee the antigen-antibody complexwa maintain the combination position, Receptor Rotation Range, Ligand Rotation Range, Twist Range were set as 0 and Distance Range was set as 1 (minimum); Correlation type was set as shape & electrostatics. The interaction energies of 429 antigen-antibody complexes were calculated and listed in S2 and S3 Tables.

### Interaction interface coordinate system generation

**Fig 2:**
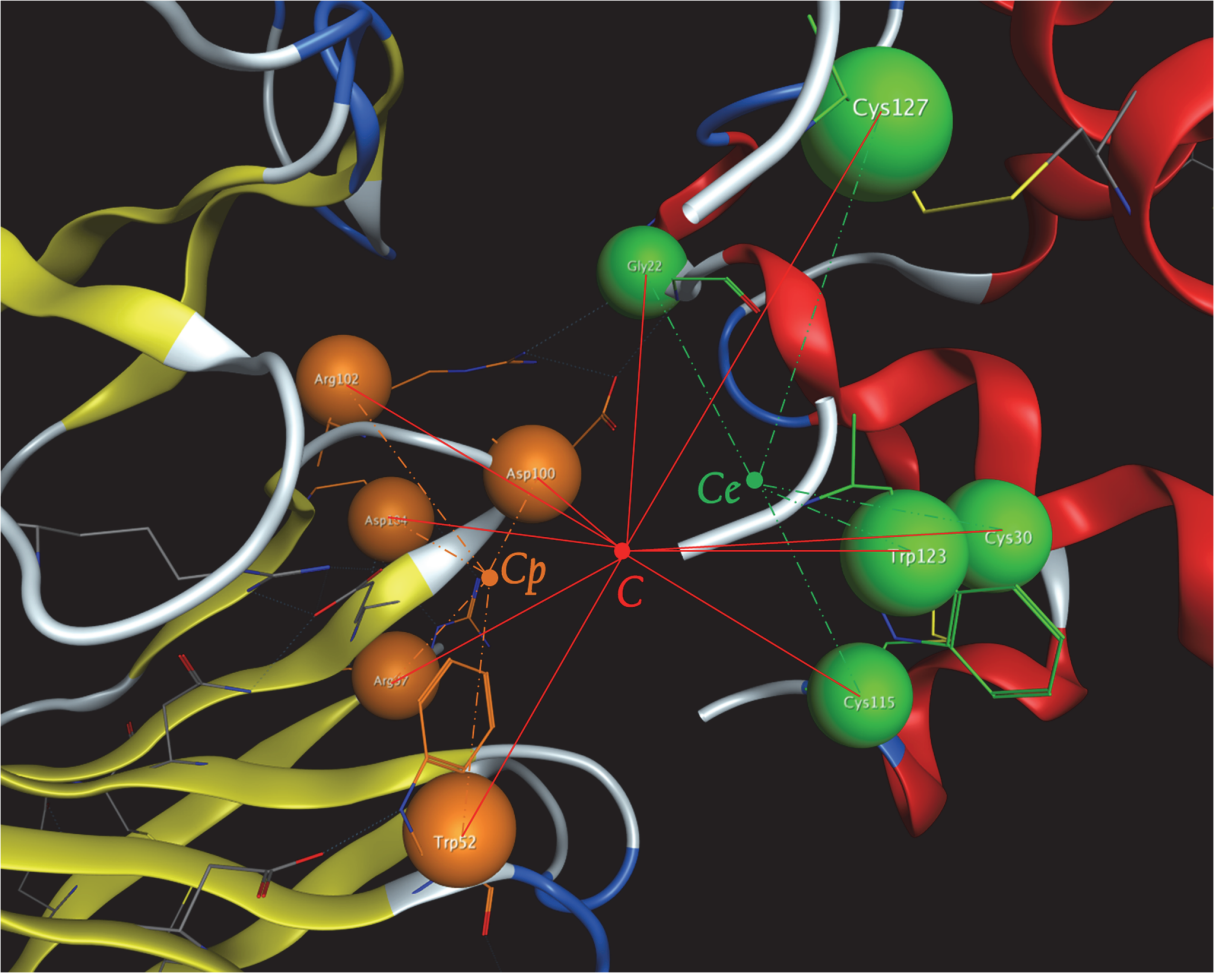
Illustration of antigen-antibody interaction coordinate system. Yellow (paratope side) and green (epitope side) balls represent the simplified point P_i_ of each residue r_i_ in the coordinate system; point C_p_ represents the geometric center of the paratope side while point C_e_ represents the geometric center of the epitope side; point C represents the geometric center of the interaction interface.

To build the protein fingerprint and EPIF, interaction coordinate system was firstly established (**Fig 2**). Here, residue r_i_ of the antigen-antibody complex was simplified as a point P_i_ by averaging its atoms’ coordinates. Then, the geometric center of epitope (C_e_) and paratope (C_p_) were calculated by averaging the coordinate of epitope residue and paratope residue respectively. Later, the geometric center (C) of interaction interface was calculated by averaging the coordinate of all the residues from both epitope side and paratope side. Based on those three points, our coordinate system can be generated.

### Protein fingerprint generation

There exist server protein description methods [35,36,32] which contains structure information mainly focusing on coordinate information, distance information and bond type/angel information of protein structures. However, previous studies illustrated that the interface features of epitope-paratope interaction may relate more to the amino acid composition, local structural and physical-chemical environment on the interaction surface [10]. It is widely reported that physical-chemical features such as hydrophobic interaction, hydrogen-bond interaction and electrostatic interaction play essential roles in the antigen-antibody interaction interface [37,38]. Here, fingerprints containing both structural features and physical-chemical environment features were established to describe the structure features of antibody in the interaction interface.

#### Structure fingerprint generation through cylinder model

**Fig 3:**
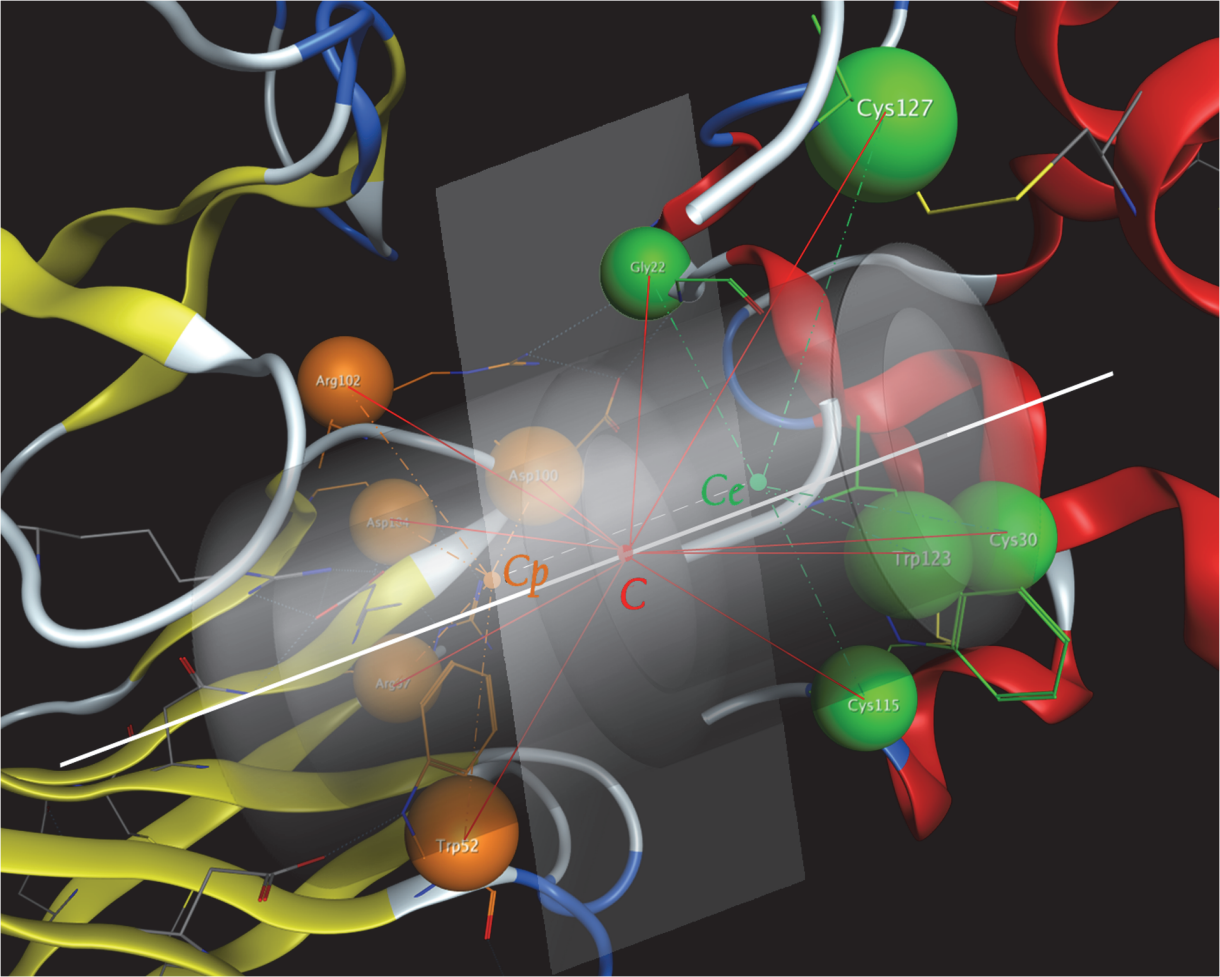
Illustration of structure fingerprint generation. Graphic definition of “virtual interaction interface” and size-defined cylinder was generated perpendicular to this virtual interaction interface.

By setting a plane through point C and perpendicular to Vector$\vec{C_{e}C_{p}}$, a virtual interaction interface was generated. This “virtual interaction interface” was set as the X-Y axis plane. With point C set as the origin, the Z axis was settled by the normal vector $\vec{n}$ of X-Y plane towards to the paratope side. Then the rotating plane was established by the X-Y-Z axis to generate the structure fingerprints. Along with a size-defined rotating plane revolving around axis Z, each of the surface residues can be punched into the certain position of the cylinder model (**Fig 3**). In order to contain enough residues in interaction interface, different plane size and grid resolution were tested. By setting 20 Å as rotating radius and 0 to 40 Å for Z axis, more than 95% of the residues on both epitope and paratope side can be projected into the structure profiles. After setting the radius pixel as 2 Å and Z axis pixel as 5 Å, a 2-dimensinal grid which contains 80 (20/2 * 40/5) bit was screened to generate the antibody protein fingerprint.

The antigen fingerprint was generated on the same system with several modifications, an idea of unit patch of residue triangle was introduced in the epitope area [39]. Unit patch of residue triangle was defined among any three surface residues where the distances for each two of them was within 4 Å, only those contain three residues were termed as epitope unit patches. For antigen structure fingerprint, the Z axis was towards to the epitope side. The averaged coordinate of three residues in a unit patches point (UP_i_) is to replace the role of residue point P_i_ in the coordinate system.

#### Physical-chemical fingerprint generation through shell structure model

**Fig 4:**
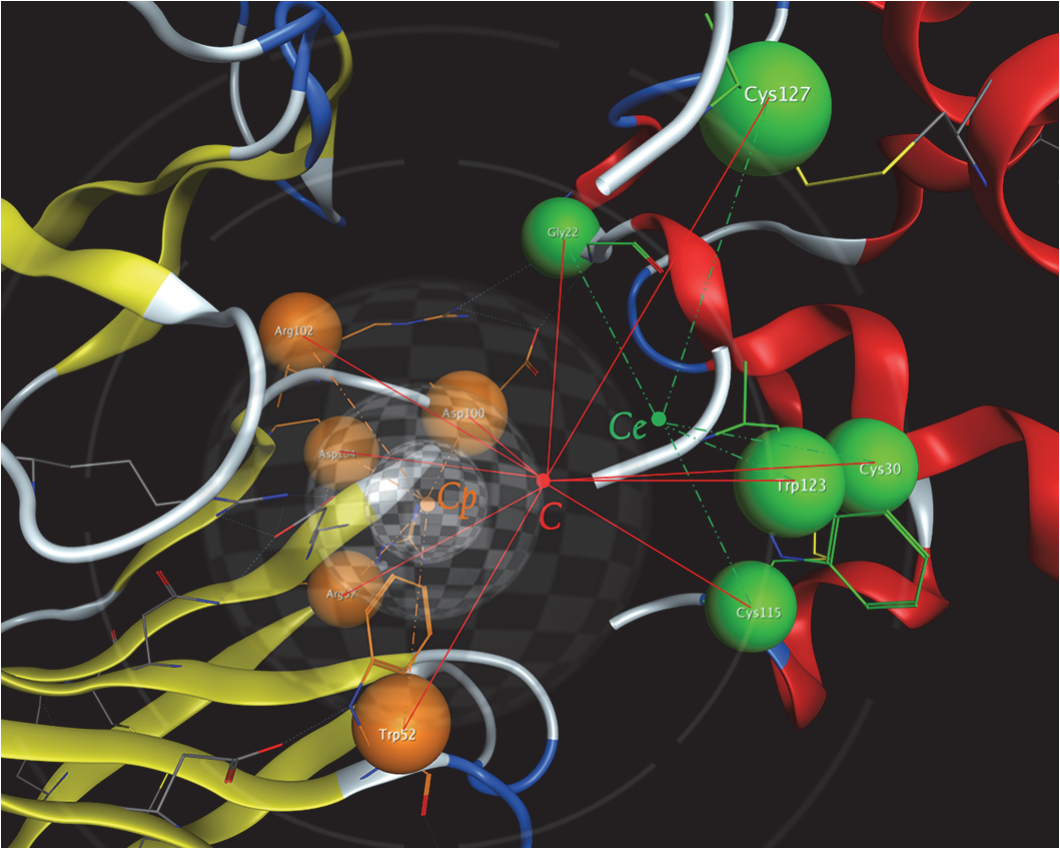
Graphic definition of shell structure model. For the shell structure model of center C_p_, 2 residues can be inputted into the second layer while 3 residues can be inputted into the third layer.

To characterize the physical-chemical environment of the protein in interaction interface, a series of shells have been generated with appropriate pixel starting from the geometric center point (C_p_ & C_e_) of each side (**Fig 4**). All neighbor residues within 20 Å from the geometric center (C_p_ & C_e_) have been counted [10] and can be inputted into different layers based on their geometric distances towards geometric center. By setting pixel distance as 2 Å, the encoding array of each physical-chemical property contains 10 independent bits. Three sets of values describing the physical-chemical properties including hydrophobic interaction, hydrogen-bond interaction and electrostatic interaction (ARGP820101, FAUJ880109 and FAUJ 880108) were derived from AAindex database [40] and led to a 30 length physical-chemical fingerprint. Different from the paratope side, the averaged AAindex of each unit patch of residue triangle was calculated as the physical-chemical index for each shell in epitope. After that, two 110-bits fingerprints for antigen and antibody side were generated respectively to characterize the unit patches layout and physical-chemical environment in the interaction interface.

#### Epitope-Paratope Interaction fingerprint (EPIF) generation

Antigen-antibody interaction interface is composed of residues from both antigen and antibody sides, appropriate spatial layout and interaction force will lead to a successful binding. To analyze an antigen-antibody complex, an epitope-paratope interaction fingerprint (EPIF) which contains both different interaction forces and environment information in 3-dimensional level is firstly established to demonstrate the interaction features of antigen-antibody complex.

Here, our approach expands the original idea of interaction fingerprint to make it suitable for the large amount of available antigen-antibody complexes data or complexes produced by docking into 3-Dimensional structures. Since EPIF is a bit string representing interactions between antigen and antibody, both the interaction force and interface environment have been fully take into consideration. Here, based on a new shell structure starting from geometric center C, a 15-bit interaction fingerprint of each residue can be inputted into 10 layers (*see “shell structure model”*). Thus, a 150-bit EPIF of each antigen-antibody complexes have been generated. The definition of 15 bits interaction fingerprint is given as follows:

#### Interaction fingerprint generation

EPIF contains eight different types of interaction: back bone, side chain, polar, hydrophobic, H-bond receptor, H-bond donor, Aromatic and Charged. Our algorithm is designed to determine those interactions by calculating atom distances and residue types. The first bit represents for any contact, if the first bit is 0 means all 14 remains are 0. For 6 strong interactions: back bone, side chain, polar, hydrophobic, aromatic and charged, an additional bit was followed to describe the interaction level of the certain interaction types, as formula 1 shows.

$$\left\{ \begin{array}{l} EPIF^{aa}=S_{1:15}{\left(epix\right)}^{aa}\\ EPIF_{i}^{aa}=\left[0\vee 1\right]i\in \left[1,15\right]\\ EPIF_{i}^{aa}=\{any;backbone\left(exist\right);backbone\left(strong\right);sidechain\left(e\right);\\ sidechain\left(s\right);polar\left(e\right);polar\left(s\right);hydrophobic\left(e\right);hydrophobic\left(s\right);\\ aromatic\left(e\right);aromatic\left(s\right);charged\left(e\right);charged\left(s\right);h-bond\;donor;\\ h-bond\;receptor;\} \end{array}\right.$$ 1

Here, *EPIF* ^*aa*^ represents an epitope-paratope interaction fingerprint for each epitope amino acid, which contains 15 bits for any amino acid *x* in the epitope side. Each bit can only be count as 0 or 1. For 8 interaction type sites (side 2,4,6,8,10,12,14,15), 1 means there exist at least one residue from paratope side which can form this type of interaction within distance cutoff, while 0 means the opposite. For 6 force strength identification sites (side 3,5,7,9,11,13), it can be count as 1 only when the same interaction type site defined as 1 and there are enough numbers of this type of interaction appeared around amino acid *x*, otherwise, it is count as 0. According to our statistical analysis, the number of residues within the distance cutoff of target ranged from 0 to 10 with the median as 4 in our dataset. Considering that the charged force is relatively stronger than other interaction forces, the number cutoff for charged was set as 1 while the others were set as 4. The distance cutoff of each site was set as 4 Å in our study [22].

### Proteochemometric Modeling

**Fig 5:**
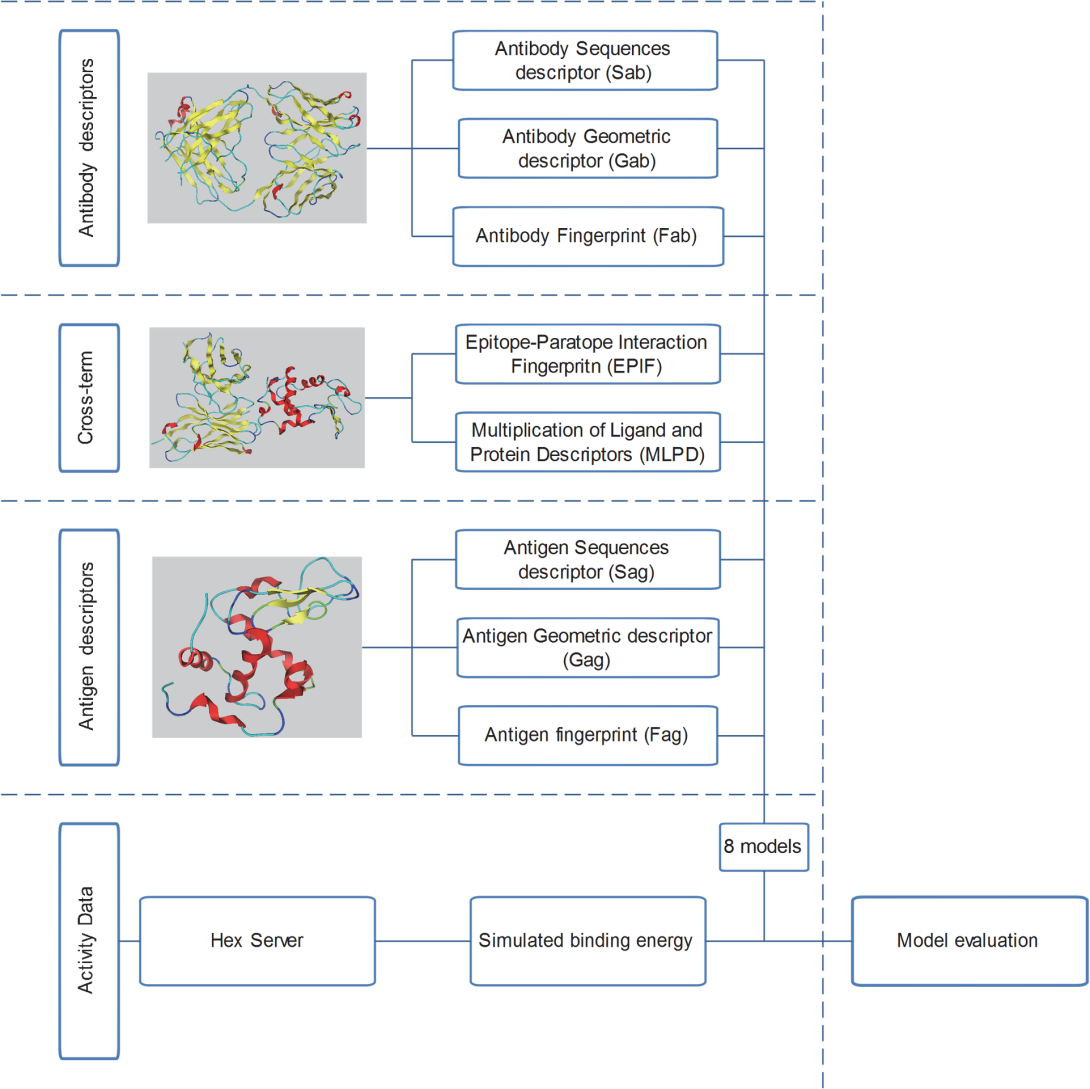
Illustration of our Proteochemometric Modeling of the antigen-antibody interaction by new protein and epitope-paratope interaction fingerprints. More detail information can be seen in *Method* & S1 Fig.

In our study, 3 Proteochemometric models were created from training set based on different combinations of fingerprints (Fab-Fag-EPIF, Fab-Fag-MLPD, Fab-Fag). All models were implemented in SMOreg of Weka (Version 3.7) by using support vector regression (SVR). The efficacy of all kernels was assessed by *Q* ^2^ (predictive ability) with 10-fold cross-validation, and two Kernels (Normalized Poly Kernel and RBF Kernel) were selected (Table 1). Additional 5 Proteochemometric models (Gab-Gag-EPIF, Gab-Gag-MLPD, Gab-Gag, Sab-Sag-EPIF, Sab-Sag) based on peers widely used sequence (*S*) and geometric descriptors (*G*) [32] with two selected kernels were established to test the performance of our fingerprints (Table 2). Also, the cross-term was tested for both EPIF and the previous multiplication of the antigen and antibody protein descriptors. Our Proteochemometric Modeling of the antigen-antibody interaction by new protein and epitope-paratope interaction fingerprints is illustrated in **Fig 5**.

## Model Evaluation

Statistical parameters for evaluating the PCM models were defined as follows:

$$MAE=\frac{1}{n}\overset{n}{\underset{i=1}{\sum }}\left|p_{i}-t_{i}\right|=\frac{1}{n}\overset{n}{\underset{i=1}{\sum }}\left|e_{i}\right|$$ 2

$$RMSE=\sqrt{\frac{\sum_{i=1}^{n}{\left(p_{i}-t_{i}\right)}^{2}}{n}}$$ 3

$$RAE=\frac{\sum_{i=1}^{n}\left|p_{i}-t_{i}\right|}{\sum_{i=1}^{n}\left|t_{i}-\bar{t}\right|}$$ 4

$$RRSE=\sqrt{\frac{\sum_{i=1}^{n}{\left(p_{i}-t_{i}\right)}^{2}}{\sum_{i=1}^{n}{\left(t_{i}-\bar{t}\right)}^{2}}}$$ 5

MAE represents Mean Absolute Error, RMSE represents Root Mean Squared Error, RAE represents Relative Absolute Error and RRSE represents Root Relative Squared Error. *p*_*i*_ represents predicted activity data calculated by different models, *t*_*i*_ represents true activity data simulated by Hex server while $\overset{-}{t}$ represents the mean of true activity data.

## Supporting Information

S1 Fig Illustration of structure model for protein fingerprint and EPIF generation. (DOCX)

S1 Table Protein geometry descriptors of each protein structure. (DOCX)

S2 Table Training dataset. (DOCX)

S3 Table Testing dataset. (DOCX)
