# Supplementary material for: Proteochemometric Modeling of the Antigen-Antibody Interaction: New Fingerprints for Antigen, Antibody and Epitope-Paratope Interaction
Source: PLoS One. 2015 Apr 22;10(4):e0122416. doi: 10.1371/journal.pone.0122416 (PMC4406442; doi:10.1371/journal.pone.0122416)
Supplement: S1 Fig — (DOCX) [file pone.0122416.s001.docx]

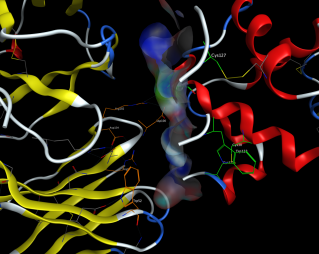

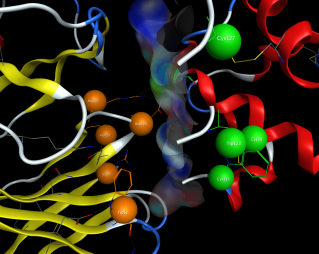

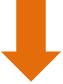

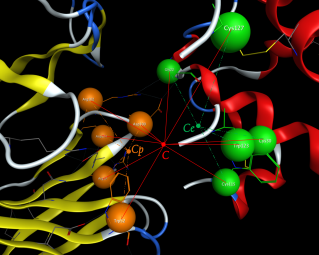

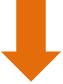

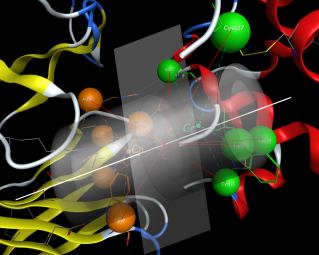

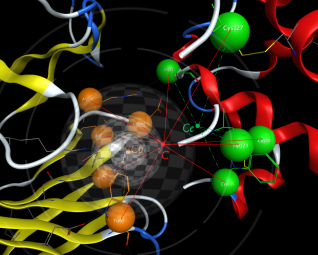

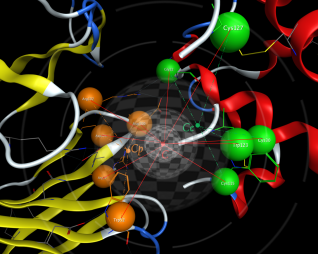

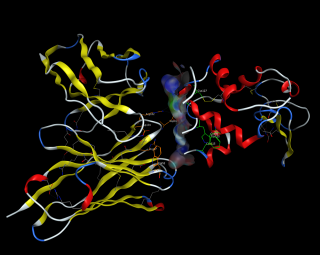

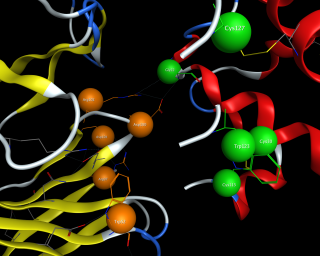

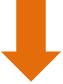

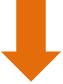

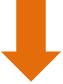

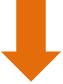

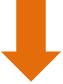


**A**

**B**

**C**

**D**

**E**

**F**

**G**

**H**

**Fig.S1 Illustration of structure model for protein fingerprint and EPIF generation.** (A) Illustration of antigen-antibody complexes, antigen on the right side and antibody on the left side. (B) Illustration of epitope-paratope interaction interface. (C) & (D) Simplified the residue in the interaction interface as a point by averaging all atoms’ coordinates. (E) Graphic definition of epitope center, paratope center and interaction center. (F) Graphic definition of coordinate system and “virtual interaction interface”, and the generation of cylinder model. (G) & (H) Illustration of shell structure model for protein fingerprint and EPIF generation.
