# Supplementary material for: Proteochemometric Modeling of the Antigen-Antibody Interaction: New Fingerprints for Antigen, Antibody and Epitope-Paratope Interaction
Source: PLoS One. 2015 Apr 22;10(4):e0122416. doi: 10.1371/journal.pone.0122416 (PMC4406442; doi:10.1371/journal.pone.0122416)
Supplement: S1 Table — (DOCX) [file pone.0122416.s002.docx]

**Table S1. Protein geometry descriptors of each protein structure^a^.**

| Bond Type | Bond Angel | Bond Angel | Dihedral Angel |
| --- | --- | --- | --- |
| C-N | N-CA(P) | CB-CA-C(A) | O-C-N(P) |
| C-N(P) | C-N-CA | CB-CA-C(I,T,V) | Chi1 g(+) |
| C-O | C-N-CA(G) | N-CA-C | Chi1 g(-) |
| CA-C | C-N-CA(P) | N-CA-C(G) | Chi1 trans |
| CA-C(G) | CA-C-N | N-CA-C(P) | Omega |
| CA-CB | CA-C-N(G) | N-CA-CB | Phi |
| CA-CB(A) | CA-C-N(P) | N-CA-CB(A) | Phi Helix |
| CA-CB(I,T,V) | CA-C-O | N-CA-CB(I,T,V) | Phi(P) |
| N-CA | CA-C-O(G) | N-CA-CB(P) | Psi |
| N-CA(G) | CB-CA-C | O-C-N | Psi Helix |

**^a^**Information was derived from PDB database (Berman, et al., 2000), Cal Ave for bond type and bond angel with the average value of Dihedral Angel ($(Minimum+Maximum)/2$) was selected as geometric descriptors. Protein structures without certain type of descriptors will be padded with 0.
