# Supplementary material for: Proteochemometric Modeling of the Antigen-Antibody Interaction: New Fingerprints for Antigen, Antibody and Epitope-Paratope Interaction
Source: PLoS One. 2015 Apr 22;10(4):e0122416. doi: 10.1371/journal.pone.0122416 (PMC4406442; doi:10.1371/journal.pone.0122416)
Supplement: S2 Table — (DOCX) [file pone.0122416.s003.docx]

**Table S2. Training dataset.**

| ID ^a^ | energy ^b^ | ID | energy | ID | energy | ID | energy |
| --- | --- | --- | --- | --- | --- | --- | --- |
| 1A14_N | -359.98 | **1R3I_C** | -235.73 | **2P42_A** | -456.19 | **3J1S_A** | -223.22 |
| 1A2Y_C | -443.92 | **1R3J_C** | -138.73 | **2P42_C** | -478.92 | **3K2U_A** | -485.16 |
| 1AHW_C | -625.45 | **1R3K_C** | -274.37 | **2P43_A** | -446.43 | **3K3Q_BC** | -688.22 |
| 1AHW_F | -721.23 | **1RI8_B** | -456.03 | **2P44_A** | -476.95 | **3KR3_D** | -366.13 |
| 1BGX_T | -701.42 | **1RJC_B** | -415.56 | **2P45_A** | -427.4 | **3L5Y_A** | -361 |
| 1BJ1_V | -67.22 | **1RZJ_GC** | -697.28 | **2P46_C** | -457.18 | **3L95_Y** | -582.64 |
| 1BQL_Y | -360.49 | **1RZK_GC** | -569.01 | **2P47_A** | -447.35 | **3LD8_A** | -623.4 |
| 1BVK_C | -381.09 | **1S5H_C** | -616.62 | **2P48_A** | -364.17 | **3LDB_A** | -526.06 |
| 1BVK_F | -390.44 | **1SLG_D** | -166.53 | **2P49_A** | -489.1 | **3LEV_A** | -497.07 |
| 1BZQ_C | -460.8 | **1STS_B** | -168.97 | **2Q8A_A** | -564.93 | **3LH2_S** | -546.98 |
| 1C08_C | -711.35 | **1STS_D** | -259.73 | **2Q8B_A** | -608.78 | **3LH2_T** | -576.6 |
| 1DQJ_C | -491.83 | **1T6V_L** | -586.8 | **2QQN_A** | -142.21 | **3LH2_U** | -535.13 |
| 1DZB_X | -649.97 | **1T6V_M** | -529.48 | **2R0L_AB** | -516.95 | **3LH2_V** | -531.04 |
| 1EGJ_A | -673.88 | **1TQB_A** | -573 | **2R29_A** | -648.99 | **3LHP_S** | -680.43 |
| 1FBI_X | -464.75 | **1TQC_A** | -551.32 | **2R56_A** | -1023.31 | **3LIZ_A** | -799.55 |
| 1FBI_Y | -457.68 | **1TZH_V** | -120.57 | **2R56_B** | -940.33 | **3LZF_A** | -327.89 |
| 1FC2_C | -413.65 | **1TZH_W** | -203.05 | **2UZI_R** | -924.99 | **3M6M_A** | -421.52 |
| 1FDL_Y | -13.2 | **1TZI_V** | -38.11 | **2VH5_R** | -1001.89 | **3M6M_B** | -651.51 |
| 1FE8_A | -461.09 | **1UA6_Y** | -685.46 | **2VOL_B** | -328.62 | **3MA9_A** | -491.76 |
| 1FE8_B | -625.86 | **1UAC_Y** | -673.48 | **2VQ1_A** | -1017.73 | **3MAC_A** | -446.84 |
| 1FE8_C | -604.69 | **1V7M_V** | -379.94 | **2VXQ_A** | -422.3 | **3MJ9_A** | -653.65 |
| 1FNS_A | -593.43 | **1V7M_X** | -416.86 | **2VXS_A** | -168.88 | **3NH7_A** | -716.42 |
| 1FSK_A | -510.34 | **1VFB_C** | -338.09 | **2VXS_D** | -17.38 | **3NH7_B** | -881.66 |
| 1FSK_D | -509.41 | **1WEJ_F** | -295.84 | **2VXT_I** | -658.01 | **3NH7_C** | -816.55 |
| 1FSK_J | -539.66 | **1XF5_PL** | -713.77 | **2W9E_A** | -246.23 | **3NH7_D** | -825.88 |
| 1G7I_C | -396.45 | **1XF5_QM** | -672.04 | **2WUB_AB** | -766.53 | **3NPS_A** | -462.87 |
| 1G7J_C | -443.46 | **1XGP_C** | -470.3 | **2WUB_CD** | -512.78 | **3O2D_A** | -212.36 |
| 1G7L_C | -370.33 | **1XGQ_C** | -371.65 | **2WUC_ABI** | -196.12 | **3P0Y_A** | -854.33 |
| 1G7M_C | -446.26 | **1XGT_C** | -415.81 | **2XQB_A** | -784.54 | **3PNW_C** | -496.65 |
| 1G9M_GC | -517.43 | **1XGU_C** | -460.58 | **2XQY_A** | -397.6 | **3PNW_F** | -453.19 |
| 1G9N_GC | -322.56 | **1XIW_AB** | -626.23 | **2XRA_A** | -758.79 | **3PNW_I** | -536.1 |
| 1GC1_G | -485.25 | **1XIW_EF** | -637.9 | **2XTJ_A** | -542.18 | **3PNW_O** | -569.15 |
| 1GC1_GC | -401.84 | **1YJD_C** | -302.01 | **2XTJ_AP** | -374.85 | **3PNW_R** | -609.72 |
| 1H0D_C | -334.33 | **1YMH_E** | -430.44 | **2XWT_C** | -703.46 | **3PNW_U** | -353.62 |
| 1HEZ_E | -339.18 | **1YMH_F** | -459.27 | **2YBR_F** | -580.02 | **3PNW_X** | -431.75 |
| 1I1A_BC | -1129.17 | **1YQV_Y** | -505.34 | **2YC1_C** | -562.26 | **3Q1S_I** | -231.31 |
| 1IC4_LY | -818.72 | **1YYL_GM** | -300.84 | **2YC1_F** | -559.19 | **3Q3G_E** | -1100.75 |
| 1IC5_LY | -810.33 | **1YYL_PS** | -387.13 | **2YSS_C** | -480.4 | **3Q3G_G** | -336.59 |
| 1IC7_LY | -736.62 | **1YYM_PS** | -347.18 | **2ZCH_P** | -568.95 | **3Q3G_I** | -537.24 |
| 1IGC_A | -383.95 | **2ADF_A** | -371.36 | **2ZNW_Y** | -659.72 | **3QA3_G** | -365.56 |
| 1IQD_C | -796.62 | **2AEP_A** | -566.25 | **2ZNW_Z** | -689.81 | **3QA3_I** | -372.85 |
| 1J1O_Y | -670.32 | **2AEQ_A** | -579.11 | **2ZNX_Z** | -773.86 | **3QA3_L** | -145.25 |
| 1J1X_Y | -701.14 | **2B2X_A** | -360.73 | **2ZU0_B** | -353.86 | **3QWO_P** | -395.09 |
| 1JPS_T | -388.06 | **2B2X_B** | -271.4 | **3A67_Y** | -775.46 | **3R1G_B** | -368.32 |
| 1JRH_I | -646.77 | **2BDN_A** | -320.02 | **3A6B_Y** | -755.26 | **3RVV_A** | -330.27 |
| 1JTO_L | -628.17 | **2BOB_C** | -120.23 | **3B2U_M** | -360.98 | **3RVW_A** | -328.09 |
| 1JTP_L | -584.56 | **2DQC_Y** | -648.57 | **3B2U_P** | -297.65 | **3RVX_A** | -355.46 |
| 1JTP_M | -600.82 | **2DQE_Y** | -676.1 | **3B2U_S** | -322.33 | **3SE8_G** | -673.31 |
| 1JTT_L | -607.49 | **2DQF_F** | -595.17 | **3B2U_V** | -372.45 | **3SE9_G** | -400.91 |
| 1K4C_C | -259.32 | **2DQG_Y** | -675.45 | **3BDY_V** | -232.05 | **3SKJ_E** | -200.15 |
| 1K4D_C | -162.39 | **2DQH_Y** | -658.05 | **3BE1_A** | -15.83 | **3SKJ_F** | -403.16 |
| 1KB5_AB | -347.71 | **2DQJ_Y** | -702.6 | **3BGF_A** | -293.37 | **3SOB_B** | -286.69 |
| 1KIQ_C | -401.89 | **2DWD_C** | -373.87 | **3BGF_S** | -232.06 | **3SQO_A** | -611.24 |
| 1KIR_C | -442.83 | **2DWE_C** | -406.97 | **3BN9_B** | -741.32 | **3SQO_AP** | -461.42 |
| 1LK3_A | -306.31 | **2EIZ_C** | -499.94 | **3C2A_LP** | -1201.35 | **3T2N_A** | -640.16 |
| 1LK3_B | -450.95 | **2EKS_C** | -528.75 | **3CVH_ABC** | -475.72 | **3T2N_B** | -484.14 |
| 1MEL_L | -647.73 | **2FD6_AU** | -276.37 | **3CVH_AC** | -557.85 | **3THM_F** | -412 |
| 1MEL_M | -604.53 | **2FD6_U** | -361.16 | **3CVH_MNO** | -429.93 | **3TJE_F** | -587.14 |
| 1MHH_F | -315.54 | **2FJG_V** | -274 | **3D85_C** | -554.62 | **3U4E_G** | -393.8 |
| 1MLC_E | -534.29 | **2GHW_A** | -767.83 | **3D85_CD** | -25.18 | **3UC0_A** | -333.08 |
| 1MLC_F | -467.79 | **2H9G_R** | -602.93 | **3D9A_C** | -385.13 | **3UC0_B** | -235.53 |
| 1N4X_L | -491.33 | **2H9G_S** | -418.14 | **3DVG_XY** | -503.64 | **3UX9_C** | -728.2 |
| 1NCA_N | -805.78 | **2HFG_R** | -404.57 | **3EOA_I** | -58.37 | **3V6O_A** | -177.94 |
| 1NCB_N | -559.22 | **2HVJ_C** | -209.3 | **3EYO_B** | -743.38 | **3V6O_B** | -330.03 |
| 1NCC_N | -713.54 | **2HVK_C** | -259.15 | **3EYO_D** | -774.53 | **3VG9_A** | -256.05 |
| 1NCD_N | -728.39 | **2I25_L** | -515.9 | **3FRU_B** | -737.15 | **4AEI_A** | -594.28 |
| 1NDG_BC | -741.94 | **2I25_M** | -572.74 | **3FRU_D** | -774.53 | **4AEI_B** | -701.79 |
| 1NDM_BC | -613.12 | **2I26_L** | -559.38 | **3FRU_F** | -848.28 | **4AEI_C** | -658.21 |
| 1NJ9_A | -1082.33 | **2I26_M** | -569.57 | **3G04_C** | -712.37 | **4AG4_A** | -371.27 |
| 1NMC_A | -343.8 | **2I26_Q** | -495.78 | **3GB7_C** | -193.57 | **4AL8_C** | -209.25 |
| 1NSN_S | -390.02 | **2I60_GM** | -232.96 | **3GBM_AB** | -147.31 | **4DGI_A** | -134.62 |
| 1OAZ_A | -402.58 | **2I60_PS** | -386.66 | **3GBM_CD** | -214.18 | **4DKE_A** | -471.22 |
| 1OAZ_B | -390.14 | **2J4W_D** | -322.74 | **3GI8_C** | -473.42 | **4DKE_B** | -567.2 |
| 1OB1_C | -452.82 | **2J5L_A** | -193.03 | **3GI9_C** | -570.73 | **4DKF_A** | -504.01 |
| 1OB1_F | -353.55 | **2J6E_AB** | -557.88 | **3GRW_A** | -537.33 | **4DKF_B** | -721.31 |
| 1OP9_B | -702 | **2NLJ_C** | -590.79 | **3H3B_A** | -581.69 | **4DN4_M** | -466.81 |
| 1ORS_C | -331.9 | **2NR6_A** | -198.06 | **3H3B_B** | -640.26 | **4DTG_K** | -407.25 |
| 1OTS_B | -272.25 | **2NR6_B** | -111.96 | **3H3P_S** | -433.18 | **4ETQ_C** | -211.34 |
| 1P2C_F | -320.61 | **2NXY_AB** | -600.7 | **3H3P_T** | -614.63 | **4F2M_E** | -541.55 |
| 1PKQ_J | -436.36 | **2NXZ_AB** | -401.18 | **3H42_AB** | -323.53 | **4F2M_F** | -424.98 |
| 1QFU_A | -495.87 | **2NY1_AB** | -669.43 | **3HI6_A** | -469.98 | **4FP8_A** | -47.91 |
| 1QFU_AB | -138.09 | **2NY3_AB** | -387.72 | **3HI6_B** | -528.6 | **4FQI_AB** | -470.86 |
| 1QKZ_AP | -371.12 | **2NY4_AB** | -488.61 | **3HMX_A** | -629.46 | **4FQJ_A** | -377.31 |
| 1QLE_AB | -45.17 | **2NY5_GC** | -639.07 | **3IDX_G** | -256.07 | **4GMS_E** | -378.93 |
| 1QLR_A | -946.37 | **2NY6_AB** | -755.23 | **3IGA_C** | -127.07 | **4HKX_E** | -513.46 |
| 1QLR_C | -889.2 | **2NY7_G** | -89.59 | **3IU3_J** | -501.06 |  | |

**^a^**ID represented the PDB id with antigen chain name.

**^b^**Interaction energy was simulated by Hex (Macindoe, et al., 2010).
