# Supplementary material for: Proteochemometric Modeling of the Antigen-Antibody Interaction: New Fingerprints for Antigen, Antibody and Epitope-Paratope Interaction
Source: PLoS One. 2015 Apr 22;10(4):e0122416. doi: 10.1371/journal.pone.0122416 (PMC4406442; doi:10.1371/journal.pone.0122416)
Supplement: S3 Table — (DOCX) [file pone.0122416.s004.docx]

**S3 Table. Testing dataset**

| ID ^a^ | energy ^b^ | ID | energy | ID | energy | ID | energy |
| --- | --- | --- | --- | --- | --- | --- | --- |
| 1AR1_AB | -316.16 | **1OAK_A** | -558.1 | **2HJF_C** | -453.85 | **3M6M_C** | -636.95 |
| 1DZB_Y | -660.33 | **1OSP_O** | -214.59 | **2IFF_Y** | -255.85 | **3MXW_A** | -958.97 |
| 1EO8_A | -404.56 | **1P2C_C** | -428.65 | **2ITD_C** | -177.94 | **3NGB_A** | -716.99 |
| 1EXU_B | -984.13 | **1PKQ_E** | -250.05 | **2JEL_P** | -413.87 | **3NGB_D** | -586.66 |
| 1EZV_E | -377.24 | **1QLE_B** | -492.85 | **2NY0_AB** | -624.78 | **3PGF_A** | -458.14 |
| 1FSK_G | -525.49 | **1R3L_C** | -220.86 | **2NY2_AB** | -588.91 | **3PNW_L** | -492.48 |
| 1G7H_C | -386.15 | **1RJL_C** | -46.44 | **2NY5_G** | -497.36 | **3Q3G_L** | -316.51 |
| 1G9M_G | -207.2 | **1SQ2_L** | -639.26 | **2P46_A** | -425.98 | **3QA3_E** | -533.48 |
| 1I1A_BCD | -459.33 | **1SY6_A** | -520.76 | **2VQ1_E** | -1092 | **3QWO_C** | -466.45 |
| 1J1P_Y | -689.76 | **1TPX_A** | -537.59 | **2W0F_C** | -86.14 | **3RJQ_A** | -857.52 |
| 1JHL_A | -500.44 | **1UJ3_C** | -547.74 | **2YBR_C** | -616.93 | **3RU8_X** | -483.06 |
| 1JTO_M | -488.48 | **1XGR_C** | -313.47 | **2YBR_I** | -598.85 | **3SO3_A** | -840.16 |
| 1JV5_A | -417.36 | **1XIW_A** | -705.28 | **2ZNX_Y** | -698.69 | **3U30_D** | -337.77 |
| 1KIP_C | -369.95 | **1YYM_GM** | -375.84 | **3A6C_Y** | -733.17 | **3U7Y_G** | -1004.59 |
| 1MHH_E | -442.22 | **1ZTX_E** | -468.69 | **3C2A_MQ** | -1206.49 | **3UX9_A** | -723.63 |
| 1MHP_A | -639.15 | **1ZV5_L** | -622.96 | **3FFD_P** | -545.28 | **4ALA_C** | -235.43 |
| 1MHP_B | -689.26 | **2DD8_S** | -422.38 | **3GBN_AB** | -323.86 | **4F3F_C** | -522.72 |
| 1N4X_M | -532.23 | **2DQD_Y** | -649.46 | **3HFM_Y** | -416.82 | **4FP8_B** | -56.4 |
| 1NJ9_L | -1063.33 | **2DQF_C** | -638.35 | **3L5W_I** | -58.63 | **4FP8_C** | -188.17 |
| 1NL0_G | -209.81 | **2DQI_Y** | -677.43 | **3L5X_A** | -465.75 | **4FP8_D** | -64.36 |
| 1NMB_N | -309.11 | **2FJG_W** | -293.53 | **3L95_X** | -733.19 |  |  |
| 1NMC_N | -394.72 | **2GHW_C** | -635.32 | **3LHP_T** | -386.04 |  |  |

**^a^**ID represented the PDB id with antigen chain name.

**^b^**Interaction energy was simulated by Hex (Macindoe, et al., 2010).

Berman, H.M.*, et al.* The Protein Data Bank. *Nucleic Acids Res* 2000;28(1):235-242.

Macindoe, G.*, et al.* HexServer: an FFT-based protein docking server powered by graphics processors. *Nucleic Acids Res* 2010;38(Web Server issue):W445-449.
